# MAVIDOS Maternal Vitamin D Osteoporosis Study: study protocol for a randomized controlled trial. The MAVIDOS Study Group

**DOI:** 10.1186/1745-6215-13-13

**Published:** 2012-02-07

**Authors:** Nicholas C Harvey, Kassim Javaid, Nicholas Bishop, Stephen Kennedy, Aris T Papageorghiou, Robert Fraser, Saurabh V Gandhi, Inez Schoenmakers, Ann Prentice, Cyrus Cooper

**Affiliations:** 1 MRC Lifecourse Epidemiology Unit, (University of Southampton), Southampton General Hospital, Southampton, UK; 2 Oxford NIHR Musculoskeletal Biomedical Research Unit, Nuffield Department of Orthopaedics, Rheumatology and Musculoskeletal Sciences, The Botnar Research Centre, University of Oxford, Oxford, UK; 3 Academic Unit of Child Health, Sheffield Children's Hospital, University of Sheffield, Sheffield, UK; 4 Nuffield Department of Obstetrics and Gynaecology, John Radcliffe Hospital, University of Oxford, Oxford, UK; 5 Sheffield Hospitals NHS Trust (University of Sheffield), Sheffield, UK; 6 MRC Human Nutrition Research, Elsie Widdowson Laboratory, Cambridge, UK

**Keywords:** Vitamin D, cholecalciferol, supplementation, trial, osteoporosis, DXA, pregnancy, neonate

## Abstract

MAVIDOS is a randomised, double-blind, placebo-controlled trial (ISRCTN82927713, registered 2008 Apr 11), funded by Arthritis Research UK, MRC, Bupa Foundation and NIHR.

**Background:**

Osteoporosis is a major public health problem as a result of associated fragility fractures. Skeletal strength increases from birth to a peak in early adulthood. This peak predicts osteoporosis risk in later life. Vitamin D insufficiency in pregnancy is common (31% in a recent Southampton cohort) and predicts reduced bone mass in the offspring. In this study we aim to test whether offspring of mothers supplemented with vitamin D in pregnancy have higher bone mass at birth than those whose mothers were not supplemented.

**Methods/Design:**

Women have their vitamin D status assessed after ultrasound scanning in the twelfth week of pregnancy at 3 trial centres (Southampton, Sheffield, Oxford). Women with circulating 25(OH)-vitamin D levels 25-100 nmol/l are randomised in a double-blind design to either oral vitamin D supplement (1000 IU cholecalciferol/day, n = 477) or placebo at 14 weeks (n = 477). Questionnaire data include parity, sunlight exposure, dietary information, and cigarette and alcohol consumption. At 19 and 34 weeks maternal anthropometry is assessed and blood samples taken to measure 25(OH)-vitamin D, PTH and biochemistry. At delivery venous umbilical cord blood is collected, together with umbilical cord and placental tissue. The babies undergo DXA assessment of bone mass within the first 14 days after birth, with the primary outcome being whole body bone mineral content adjusted for gestational age and age. Children are then followed up with yearly assessment of health, diet, physical activity and anthropometric measures, with repeat assessment of bone mass by DXA at age 4 years.

**Discussion:**

As far as we are aware, this randomised trial is one of the first ever tests of the early life origins hypothesis in human participants and has the potential to inform public health policy regarding vitamin D supplementation in pregnancy. It will also provide a valuable resource in which to study the influence of maternal vitamin D status on other childhood outcomes such as glucose tolerance, blood pressure, cardiovascular function, IQ and immunology.

## Background

### Osteoporosis: Epidemiology and impact

Osteoporosis is a skeletal disorder characterised by low bone mass and micro-architectural deterioration of bone tissue, which predisposes to fracture [1]. These fractures typically occur at the hip, spine and wrist. It has been estimated that the remaining lifetime risk of fracture at age 50 years approaches 50% among women and 20% among men [2]. In the UK, the annual cost to the National Health Service of managing osteoporotic fracture is £1.7 billion, with about 80% of this figure attributable to hip fracture [3].

### Early growth and later risk of fracture

Bone mass (a composite of size and volumetric density) increases through early life and childhood/adolescence to a peak in early adulthood. The magnitude of this "peak bone mass" is a strong predictor of later osteoporosis risk [4]. Cohort studies in adults from the UK, USA, Australia and Scandinavia have shown that those who were heavier at birth or in infancy have a greater bone mass [5-8] and a reduced risk of fracture [9] in later life. Mother-offspring studies have revealed that factors such as maternal lifestyle, body build, diet and physical activity during pregnancy may all influence offspring bone mineral accrual [10-12].

### Maternal vitamin D insufficiency and offspring bone mineral accrual

The specific micronutrient that appears to have the greatest individual contribution, certainly for the developed world, is vitamin D. There are very few data on vitamin D levels in pregnant women across a sample representative of the UK; the available studies, however, suggest that vitamin D insufficiency is common in this group. In a Southampton pregnancy cohort, composed of white Caucasians, 31% had concentrations of circulating 25(OH)-vitamin D lower than 50 nmol/l and 18% less than 25 nmol/l [13]. A 2010 US study of a population representative of the national demographic distribution revealed that 80% of black pregnant women had levels less than 50 nmol/l; the figures for Hispanic and white pregnant women were 45% and 13% respectively [14]. In Asian cohorts in the northern hemisphere the burden of insufficiency is even higher [15-19], possibly reaching 90% or greater: A study of non-pregnant South-Asian women in the North of England, many of whom were of child-bearing age, demonstrated that 94% had circulating levels of 25(OH)-vitamin D < = 37.5 nmo/l and 26% < = 12.5 nmol/l [20]. As the main source of vitamin D for most people is synthesis in the skin under the influence of UVB radiation from sun light exposure, ethnicity, skin pigmentation, covering and northerly latitudes (as in UK) are all major risk factors for insufficiency [21].

In the Southampton cohort [13], lower concentrations of serum 25(OH)-vitamin D in mothers during late pregnancy were associated with reduced whole-body BMC and BMD in children at age 9 years, independently of social class, diet and body size. Estimated exposure to ultraviolet B radiation during late pregnancy and the maternal use of vitamin D supplements both predicted maternal 25(OH)-vitamin D concentration and childhood bone mass. Adjunctive evidence supporting a role for maternal vitamin D status was obtained in the Southampton Women's Survey (SWS), where maternal vitamin D concentrations correlated positively with neonatal bone mass [22]. A 2009 study of the Avon Longitudinal Study of Parents and Children [23] demonstrated a positive association between ambient ultraviolet B radiation in pregnancy and offspring BMC at 9 years, further supporting the notion that maternal vitamin D status is an important determinant of offspring bone development. Furthermore, high resolution ultrasound measurements of pregnancies in the SWS cohort have revealed that changes in distal femoral morphology (increased splaying of the metaphysis relative to its length, reminiscent of changes observed in postnatal rickets) are associated with low circulating concentrations of 25(OH)-vitamin D in the mother as early as 19 weeks gestation [24]. The effect of maternal 25(OH)-vitamin D status appeared to be partly mediated via concentrations of venous umbilical cord calcium, suggesting that placental calcium transfer to the fetus may be a critical step in these associations. Indeed mRNA expression of an active placental calcium ATPase positively predicted offspring BMC in SWS neonates [25].

### Previous trials of vitamin D supplementation in pregnancy

There have been several, mainly small, intervention studies examining this issue [16-19,26,27], but only one has examined bone mass at birth [28]. In this study bone mass of offspring born to 19 Asian mothers who had taken 1000 international units of vitamin D daily through pregnancy was compared with that of babies born to 45 control mothers using single photon absorptiometry. The small participant numbers and technique used to assess bone mineral mean that it is difficult to draw any definite conclusions from negative results of this study. These studies suggest that daily doses of 1000 IU cholecalciferol or higher doses as a bolus appear to be safe, at least in the short term. National guidance has been rather conflicting over recent years, with both Cochrane [29] and NICE (CG62 http://www.nice.org.uk/nicemedia/live/11947/40115/40115.pdf) reviews concluding that there was insufficient evidence on which to base a firm recommendation; the latter suggested supplementation with 400 IU daily for high risk groups. The UK Scientific Advisory Committee on Nutrition (SACN), building on previous recommendations from the Committee on Medical Aspects of Food and Nutrition Policy (COMA)[30], reviewed the area in 2007 http://www.sacn.gov.uk/pdfs/sacn_position_vitamin_d_2007_05_07.pdf and DH, in *The Pregnancy Book *http://www.dh.gov.uk/prod_consum_dh/groups/dh_digitalassets/@dh/@en/@ps/@sta/@perf/documents/digitalasset/dh_107667.pdf now advise all women to take 400 IU cholecalciferol daily. It is unclear how well these guidelines are currently followed in the UK however, but anecdotal evidence suggests that take up has been low.

## Aim

To test the hypothesis that vitamin D supplementation during pregnancy of women who have low levels of vitamin D will result in improved neonatal bone mineral content when compared with offspring of women who were not supplemented.

### Study design

MAVIDOS is a randomised, double-blind, placebo controlled trial, comparing 1000 IU cholecalciferol orally daily with matched placebo in pregnant women from 14 weeks gestation until delivery of the baby, with neonatal whole body bone mineral content, assessed by DXA, as the primary outcome.

### Methods (Figure 1)

**Figure 1:**
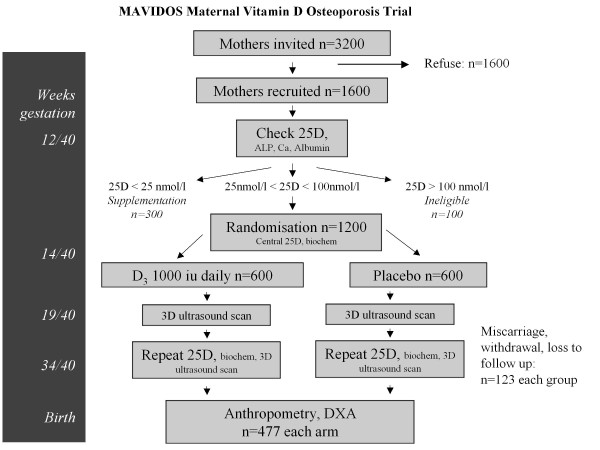
**MAVIDOS trial outline, showing expected recruitment and withdrawal through study**. Timings are given relative to the 40 week normal gestation.

#### Recruitment

Information about the trial is displayed as posters in general practice surgeries and maternity hospital waiting areas. An information sheet outlining the study is sent out to each woman with the letter for the screening appointment for nuchal lucency or initial dating scan (at around 12 weeks gestation). Information is translated into other languages where possible, to maximise participation by ethnic minorities. A research nurse approaches the women whilst they wait for the screening ultrasound scan, involving an interpreter if required and available. If informed consent for participation in the study is gained, a blood sample for 25(OH)-vitamin D is taken along with the standard Down's screening bloods or alone if only a dating scan is being performed.

#### Screening 25(OH)-vitamin D concentration

Circulating 25(OH)-vitamin D is measured locally; all three laboratories (Southampton, Oxford, Sheffield) participate in DEQAS vitamin D quality assurance system http://www.deqas.org/. Women with circulating levels of less than 25 nmol/l are excluded from the trial (pre-randomisation) and results are forwarded to their general practitioners in order that vitamin D supplementation can be prescribed. The general practitioner is advised to start either one Healthy Start Vitamin daily if available, or a prescribed calcium and vitamin D supplement yielding the same daily dose of cholecalciferol (400 IU). The general practitioner is also advised to recheck the 25(OH)-vitamin D level at the 34 week blood tests. Likewise, women with circulating levels of greater than 100 nmol/l are excluded. Those women whose circulating 25(OH)-vitamin D falls between 25 nmol/l and 100 nmol/l are randomised to an oral vitamin D supplement (1000 IU cholecalciferol per day) or matched placebo from 14 weeks (or as soon as possible after obtaining 25(OH)-vitamin D status if participant is enrolled at greater than 12 weeks gestation) to delivery in a double-blind randomised trial design. At the initial recruitment, participants are given a urine collection bottle and instructions on collecting a second void urine (for bone turnover markers) to bring to the 14 week assessment.

#### 14 week assessment

Women recruited are assessed at 14 weeks gestation (or as soon as possible after obtaining 25(OH)-vitamin D status up to 17 weeks if enrolled at greater than 12 weeks gestation), either at home or in the research clinic. Here, study medication is issued and an interviewer-led questionnaire is administered to gather information on parity, baseline demographics, smoking, alcohol intake, calcium and vitamin D dietary intake, exercise, health, sunlight exposure and medication. A subgroup of women (n = 50) are invited to wear a sunlight exposure badge, incorporating a polysulphone film, which changes its composition according to ultra-violet exposure, for one week to validate the sunlight exposure questionnaire data. Height, weight, skin fold thickness (triceps, biceps, subscapular and suprailiac) and grip strength are measured. Inter-observer variation (IOV) studies will be performed in each centre, after central training according to the study protocol. A further blood sample is taken and plasma is stored at -80°C for measurement of 25(OH)-vitamin D, vitamin D binding protein (DBP), calcium, bone specific alkaline phosphatase and albumin centrally (MRC Human Nutrition Research, Cambridge, UK) at the end of the study. If consent for genetic analyses is obtained, blood pellets for later DNA extraction are stored. Where the baby's father attends at either the 14 or 34 week visit, their height is measured.

#### Randomization and issue of Investigational Medicinal Product (IMP)

The IMP and matched placebo are manufactured by Bilcare GCS (Europe) Ltd, Waller House, Elvicta Business Park, Crickhowell, Powys NP8 1DF, UK. The manufacturers had no role in the trial other than supply of the randomised IMP. The IMP capsule contains 1000 international units of cholecalciferol (vitamin D3), with excipients matched to the placebo capsule. The dose was chosen with the aim of achieving repletion without causing supranormal circulating levels of 25(OH)-vitamin D [31]. The medication is blister packed in a single box for each woman for the duration of pregnancy. Study medication (active/placebo) is supplied to the local pharmacy pre-randomised by the manufacturer (1:1, unstratified by centre) and sequentially numbered for storage and dispensing. Code break envelopes are supplied to the lead pharmacist, but are not available to the investigative team. Emergency code break access is available through the local principal investigator and on-call pharmacist. A single pack for each participant is issued sequentially (containing all pills for duration of the study). Each pack is individually prescribed for each participant. The trials pharmacist allocates a pack to that prescription, documenting both the pack number and the MAVIDOS participant ID; these are checked again by the research nurse on collection, and documented in the participant's notes; the medication pack comes with a tear-off adhesive label, which is placed in the participant's notes as an added safeguard against errors in pack allocation. The research nurse collects the medication pack for all participants attending that day and issues to the participants directly. A clear sticker is applied to the front of participant obstetric notes, alerting clinicians to the mother's enrolment in the study. An information sheet and one copy of the consent form are filed in the participant's notes. The mother (with a copy for the father) is given contact details for the study coordinator and the research nurse, and asked to inform them immediately of commencement of labour. A second urine collection bottle and instructions on collecting a second void urine are issued, to be brought to the 34 week assessment.

#### Anomaly/3D ultrasound scan at 18-21 weeks gestation

Participants return at 18-21 weeks gestation for a high resolution 3D ultrasound scan to obtain detailed measurements of the pregnancy http://www.bmus.org/policies-guides/SoR-Professional-Working-Standards-guidelines.pdf. The NHS anomaly scan is also performed at this visit by a MAVIDOS ultrasonographer. Should any abnormalities be identified, these participants are referred to their local fetal medicine department for further management and may be excluded from this study. These findings will also be recorded as adverse events and thus may inform potential new hypotheses regarding the action of vitamin D in pregnancy. A pill count is performed to assess compliance with the study medication.

#### 34 week visit and 3D ultrasound scan

At 34 weeks gestation (up to before admission for delivery) the mothers return to the research clinic, when repeat blood samples for 25(OH)-vitamin D, DBP, calcium, bone specific alkaline phosphatase and albumin are taken. Assessments of maternal anthropometry, calcium and vitamin D intake, smoking, alcohol, exercise, medications and health are also repeated. Compliance with the study medication is assessed by pill count. A repeat growth and 3D ultrasound scan is performed [32]. Any abnormalities noted on the scan are recorded. The mother is given an information sheet about the neonatal DXA assessment at this visit. Mothers receive standard antenatal care and any woman found to be hypercalcaemic (serum calcium > 2.75 mmol/l) will be followed-up and managed appropriately.

#### Admission to hospital for labour

The study coordinator/research nurse is informed of any woman entering labour by the NHS midwives, or by the woman/partner themselves. Obstetric history is recorded. The attending midwives collect venous umbilical cord blood (including samples for genetic analyses) and placental and umbilical cord tissue samples at delivery. After ensuring that the hips have been assessed and cleared by a paediatrician, a research nurse measures the baby's anthropometric indices (length, weight, skinfolds, head and abdominal circumference) and arranges an appropriate time for the baby to undergo bone density assessment by DXA. She also issues the mother with cotton wool balls, a collection pot and instructions on collecting a urine sample from the baby. The urine sample is then brought to the DXA appointment.

#### Neonatal DXA assessment

The reliability of DXA measurements in neonates has been well documented in the Southampton Women's Survey [11]. The baby is pacified, fed, fully undressed and then swaddled in a towel before placement on the densitometer [Hologic Discovery instrument using paediatric software (Hologic Inc., Bedford, MA, USA)]. The scanner is calibrated against a spine phantom every day together with daily quality assurance and periodic step-wedge calibration all performed as per manufacturer's instructions (Hologic Inc., Bedford, MA, USA). If possible the baby is scanned whilst still an inpatient, otherwise the mother returns within 14 days for assessment. Whole body and lumbar spine bone area, bone mineral content and bone mineral density are measured. Appropriate cross-calibration of DXA scanners between the 3 centres will be made with neonatal phantoms. Information on pregnancy complications is collected from the hospital notes. The total radiation dose (0.04 mSv) is equivalent to around 2 day's exposure to background radiation in Cornwall (UK) or 7 day's exposure in other parts of the UK.

#### Maternal DXA assessment

To enable assessment of the influence of vitamin D insufficiency and repletion on the maternal skeleton, consecutive participants are asked for consent to perform additional DXA assessment of the mother, using the Hologic Discovery instrument, at the neonatal DXA visit. An information sheet about this is given to the mother at the 34 week visit, making clear that this assessment is additional to the core trial protocol, so women are free to decline without their participation in the core trial protocol being affected. Whole body, lumbar spine and both hips are measured. The radiation dose for these scans is: whole body 4.2 μSv, lumbar spine 4.4 μSv and each hip 2.4 μSv (Hologic Inc., Bedford, MA, USA)

#### Paediatric follow-up

The children are assessed at 1, 2, 3 and 4 years of age to characterise diet, health, exercise and anthropometric measurements. At 4 years the children undergo a repeat DXA assessment of bone mass at whole body, lumbar spine, and additionally at hip sites. This will utilise the same DXA instrument as for the birth measure. If the family has moved to another location, attempts will be made to follow the child up and invite them back for annual follow-up and 4 year DXA assessment. Table 1 summarises the data collected at each time point.

**Table 1:** Summary of trial events

	12 weeks	14 weeks	34 weeks	Delivery	0-14 days
Screening	√				

Consent	√				

Maternal height		√	√		

Maternal weight		√	√		

Maternal skin folds		√	√		

Maternal grip strength		√	√		

Maternal medication		√	√		

Maternal supplements		√	√		

Maternal diet		√	√		

Maternal health		√	√		

Family health	√	√	√		

Ultrasound scan	√		√		

Maternal blood for 25(OH)-vitamin D, Ca, Alb, bALP, DBP Blood pellet	√	√	√		

Maternal urine sample for bone markers		√	√		

Umbilical venous blood and Placental tissue				√	

Infant urine sample				√	

Infant length				√	

Infant weight				√	

Infant skinfolds				√	

Infant DXA					√

### Inclusion and exclusion criteria

#### Inclusion criteria

• Less than 17 weeks gestation at first assessment (based on last menstrual period (LMP) and dating scan)

• Serum 25(OH)-vitamin D concentration is 25-100 nmol/l at nuchal fold/dating scan (10 to 17 weeks gestation)

• Aged over 18 years

• Singleton pregnancy

• Aiming to give birth at local maternity hospital

#### Exclusion criteria

• Known metabolic bone disease or chronic disease known to be associated with bone abnormalities

• Current medication likely to interfere with intrauterine growth (corticosteroids, enzyme-inducing anticonvulsants, PTH, bisphosphonates, more than 6 months GnRH analogues)

• Current use of supplement containing vitamin D with daily dose > 400 IU

• Foetal physical anomalies on the initial or 18-21 weeks scan (likely to influence bone measurement by DXA or interfere with skeletal development)

• Inability to provide informed consent or comply with trial protocol

• History of renal stones, hyperparathyroidism, hypercalciuria

• A diagnosis of cancer in the last 10 years (other than basal cell carcinoma)

• Serum calcium > 2.75 mmol/l

### Timing and inclusion

Many women do not know accurately when they conceived. Thus gestation based on LMP and that on dating ultrasound may differ. Therefore women coming for assessment at 12 weeks may find that they are actually several weeks earlier or later than this.

• Dating scans may be from 8 weeks; inclusion for trial is from 8 weeks to 17 weeks gestation.

• Nuchal lucency scanning is not usually possible before 11 weeks http://www.bmus.org/policies-guides/SoR-Professional-Working-Standards-guidelines.pdf, so any women in earlier gestation is asked to return at 12 weeks for a repeat scan, and study medication issue will still be at 14 weeks.

• Women found to be at later gestation (between 13 and 17 weeks) are included and blood taken at the initial visit by the obstetric team or MAVIDOS research nurse. Medication is issued at a second visit as soon as possible after 25(OH)-vitamin D status is known.

• 14 week visits are acceptable up to anomaly scan; this scan must be between 18 and 21 weeks to comply with NHS timing.

• 34 week visit acceptable up till delivery.

• Target for 34 week scan is 33 to 36 weeks.

• Postnatal DXA to be performed within 14 days after delivery.

### Sample size and power calculation

Sample size was estimated [Stata V11.0 (Statacorp, Texas, USA)] using the results from a previous Southampton-based mother-offspring cohort (Princess Anne Hospital Study [13]), in which a difference of 0.42 sd in whole body bone mineral content was found between the offspring of mothers who had been vitamin D deficient and those of mothers who had been vitamin D replete during pregnancy. To detect 50% of the difference in whole body bone mineral content at birth between offspring of mothers who were deficient in vitamin D versus those replete in pregnancy (0.21 sd or 3.5 g), at the 5% significance level with 90% power, would require recruitment of 477 infants to each arm. Allowing for the expected miscarriage rate and 20% drop-out rate, we would need to recruit 600 mothers to each arm. We estimate the prevalence of vitamin D between 25 and 100 nmol/l in the Southampton pregnant population to be around 80%, suggesting initial screening of 1,600 women will be required. Pilot data suggest that around 50% of women who are approached will agree to participate in the study; initial invitations will therefore be sent to 3,200 women.

Around 3500 women attend the Princess Anne Hospital, Southampton for Down's screening/dating scan each year, as part of a national programme involving collection of serum samples and an ultrasound scan. Previous experience with the Southampton Women's Survey suggests a response rate of around 50%, with around 85% retention through to term pregnancy, with subsequent DXA assessment of the neonate. Further samples of 3000 incident pregnancies will be available in each of the other two centres to participate in the trial (Professor N Bishop, University of Sheffield; Dr S Kennedy, University of Oxford).

### Statistical analysis

It is anticipated that there will be some missing data during the trial because patients will be lost to follow-up (non-completers) or will provide incomplete data (non-compliers). To avoid consequent bias, intention-to-treat (ITT) analysis will be used, incorporating all women randomised to either treatment or placebo, regardless of whether the offspring underwent DXA assessment at birth. To fully account for participants whose offspring did not undergo neonatal DXA assessments, we will follow the framework proposed by White et al. [33] for ITT analysis that depends on making plausible assumptions about the missing data and including all participants in sensitivity analyses. Analyses will be performed using Stata V11.0 (Statacorp, Texas, USA).

### Primary outcome

Whole body bone mineral content of the neonate, adjusted for gestational age and age at neonatal DXA scan.

### Secondary outcomes

Whole body bone area, bone mineral density, and size-corrected bone mineral density (BMC adjusted for BA, length and weight), body composition adjusted for gestational age and age at DXA scan.

The primary analysis will not be stratified by centre (Southampton, Sheffield, Oxford) or ethnicity. Tests for interactions with offspring gender, season of birth, maternal parity, recruitment centre and ethnicity will also be performed. Separate analyses will be based on those participants who completed the protocol, secondly on those who complied with treatment, thirdly on those who demonstrated a rise in maternal 25(OH)-vitamin D concentration and fourthly on those whose initial 25(OH)-vitamin D level was below 50 or 75 nmol/l.

### Statistical tests

The primary outcome is neonatal bone mineral content as measured by DXA within two weeks following birth, presented as a continuous variable. The primary comparison is treatment (1000 iu cholecalciferol daily) versus control (placebo capsule daily). Linear regression modelling (ANOVA) will be used to describe the association of treatment on outcome. Student's t-tests may also be used to compare groups in secondary analyses.

### Ethical considerations

The study involves the participants undergoing various procedures with which they may not be familiar. Detailed information sheets are given to the participants and they have opportunities to discuss any concerns in detail with study personnel. The infant DXA assessments are associated with a low dose of radiation exposure equivalent to 2 days background radiation in Cornwall (UK) or 7 days in other parts of the UK. Data from the Princess Anne Cohort study showed a small excess of atopic asthma in children born to mothers with the highest levels of vitamin D in pregnancy [34]. However the numbers were lower than expected from the general population and confidence intervals wide. No associations between atopy in infancy and high levels of maternal vitamin D have been shown in the Southampton Women's Survey, and other studies have suggested neutral or negative associations [35-37]. The dose of vitamin D supplementation has been chosen to bring women just into the normal range, to avoid elevating it to supranormal levels.

### Reporting of adverse events

#### Anticipated Adverse Events

The development of some infrequent pregnancy-associated complications are anticipated to arise spontaneously during the study (ie cholestasis of pregnancy, pregnancy induced hypertension, gestational diabetes, pre-eclampsia, eclampsia, premature labour or delivery, instrumental delivery, caesarean section, miscarriage or stillbirth http://guidance.nice.org.uk/CG62/NICEGuidance/pdf/English).

#### Adverse drug reactions and adverse drug events

Any serious adverse event which is felt in any way to be related to the IMP is immediately reported (verbally or in writing) to the sponsor. This is followed by a detailed written report on the event. The local Principal Investigator decides whether to expedite reports of adverse events felt to be unrelated to the IMP. A record of all serious adverse events is kept in the trial master file regardless of whether reported within 24 hours to the sponsor.

The sponsor keeps detailed records of all adverse events relating to a clinical trial which are reported to them by the investigators for the trial. These records may be sent to the licensing authority if required.

#### Suspected unexpected serious adverse reactions

The sponsor ensures that all relevant information about a suspected unexpected serious adverse reaction which occurs during the course of the trial and is fatal or life-threatening is reported as soon as possible to the MHRA, and the relevant ethics committee/data monitoring committee. This is done not later than seven days after the sponsor was first aware of the reaction. Any additional relevant information is sent within eight days of the report.

The sponsor ensures that a suspected unexpected serious adverse reaction which is not fatal or life-threatening is reported as soon as possible, and in any event not later than 15 days after the sponsor is first aware of the reaction, to the MHRA and the relevant ethics committee/data monitoring committee.

### Monitoring

#### Trial Steering Committee

Professor David Reid (independent chair), Ms Caroline Dore (statistician and independent observer), Professor Cyrus Cooper (chief investigator), Dr Nicholas Harvey (lead principal investigator), Professor Nick Bishop (principal investigator), Dr Stephen Kennedy (principal investigator), Dr Kassim Javaid (principal investigator), Dr Aris Papageorghiou (principal investigator), Dr Saurabh Gandhi (principal investigator), Professor Richard Eastell (independent member), Dr Zulf Mughal (independent member), Mrs Doreen Hedger (lay representative).

#### Independent Data Monitoring Committee

Professor Deborah Symmons, Professor Roger Francis, Dr Mark Philips, Dr Chris Roberts

### Regulatory Aspects

The study has received approval from MHRA, Southampton and Southwest Hampshire REC, University Hospital Southampton Trust R and D (Sponsor), UHS Data Protection Office. The IMP and placebos are manufactured in accordance with GMP regulations. The study is conducted in compliance with the Research Governance Framework for Health and Social Care, the Medicine for Human Use (Clinical Trials) Regulation 2004 and GCP. Indemnity has been provided through University Hospital Southampton NHS Foundation Trust (sponsor) and University of Southampton.

## Discussion and potential impact

As far as we know, MAVIDOS is one of the first ever tests, in humans, of the concept that an intervention in the mother during pregnancy may influence offspring bone mineral accrual. The result, positive or negative, should provide a step change in the evidence guiding current and future public health policy regarding vitamin D supplementation in pregnancy. The longer term follow up of these children will be important because it is possible that subtle effects of vitamin D supplementation, such as a reduction in allergy or asthma, will be only be detected after several years of observation. Given the current enthusiasm for vitamin D supplementation, and the possible widespread uptake, it is highly important that assessment of its effects is not limited purely to those during and immediately after pregnancy.

### Trial Status

The MAVIDOS study is ongoing.

## Abbreviations

Alb: Albumin; ALP: Alkaline phosphatise; ATP: Adenosine Tri-Phosphate; BA: Bone Area; BMC: Bone Mineral Content; BMD: Bone Mineral Density; BMUS: British Medical Ultrasound Society; BRU: Biomedical Research Unit; Ca: Calcium; COMA: Committee on Medical Aspects of Food and Nutrition Policy; DBP: Vitamin D Binding Protein; DEQAS: Vitamin D External Quality Assessment Scheme; DNA: Deoxyribonucleic Acid; DMC: Data Monitoring Committee; DXA: Dual-Energy X-ray Absorptiometry; GCP: Good Clinical Practice; GMP: Good Manufacturing Practice; GnRH: Gonadotrophin Releasing Hormone; HMSO: Her Majesty's Stationery Office; ISRCTN: International Clinical Trials Registry Number; IMP: Investigational Medicinal Product; IOV: Inter-operator Variation; IQ: Intelligence Quotient; ITT: Intention To Treat; LMP: Last Menstrual Period; MAVIDOS: Maternal Vitamin D Osteoporosis Study; MHRA: Medicines and Healthcare products Regulatory Agency; MRC: Medical Research Council; mRNA: messenger Ribonucleic Acid; NHS: National Health Service; NIHR: National Institute for Health Research; PTH: Parathyroid hormone; NICE: National Institute for Health and Clinical Excellence; SACN: Scientific Advisory Committee on Nutrition; SWS: Southampton Womens Survey; UK: United Kingdom; UHS: University Hospital Southampton NHS Foundation Trust; USA: United States of America; UVB: Ultra-violet B.

## Competing interests

The authors declare that they have no competing interests.

## Authors' contributions

All authors took part in the design of the study. NH is responsible for operational supervision of the study as lead principal investigator, and drafted the manuscript. KJ, ATP and SK are PIs at the Oxford site. NB, RF and SG are PIs investigators at the Sheffield site. IS and AP are responsible for the biochemical testing. CC is the chief investigator and has overall responsibility for the study. All authors participated in the preparation of, and approved for publication, the final manuscript.

## Note

**Funding: Arthritis Research UK, Medical Research Council, Bupa Foundation and NIHR**. ISRCTN82927713, registered 2008 Apr 11. Date first participant randomised: 2008 Oct 7
